# The Dose Comparison of Intrathecal Morphine for Postoperative Analgesia in Total Knee Arthroplasty Under Spinal Anesthesia: A Single Institute Retrospective Study

**DOI:** 10.7759/cureus.49350

**Published:** 2023-11-24

**Authors:** Promil Kukreja, Jacelyn E Peabody Lever, Hanna Hussey, Paul Piennette, Peter Nagi, Scott Mabry, Joel Feinstein, Brooke Vining, Jason Gerlak, Christopher A Paul, Hari Kalagara

**Affiliations:** 1 Anesthesiology and Perioperative Medicine, University of Alabama at Birmingham (UAB), Birmingham, USA; 2 Anesthesiology and Perioperative Medicine, University of Alabama at Birmingham School of Medicine, Birmingham, USA; 3 Orthopaedics, University of Alabama at Birmingham (UAB), Birmingham, USA; 4 Anesthesiology and Perioperative Medicine, Mayo Clinic, Jacksonville, USA

**Keywords:** post-operative analgesia, oral morphine equivalents, total knee arthroplasty, spinal anesthesia, intrathecal morphine

## Abstract

Background and purpose of the study

Intrathecal morphine (ITM) provides effective postoperative analgesia in patients undergoing total knee arthroplasty (TKA) under spinal anesthesia. However, the ideal dose at which maximal analgesic effects can be delivered with minimal side effects is not clearly known. This retrospective study is aimed to compare two different doses of ITM with respect to analgesia benefits and side effects.

Methods

This is a retrospective, descriptive, single-center study approved by the Institutional Review Board (IRB) at the University of Alabama at Birmingham. Three patient groups were selected: a control group receiving continuous adductor canal block (CCACB) under spinal anesthesia, and two experimental groups receiving single-dose adductor canal block (SSACB) under spinal anesthesia with either 100 mcg or 150 mcg of ITM. The sample size included 75 patients (25 per group) who were 18 years and older, American Society of Anesthesiology (ASA) class 1-3 who were undergoing primary TKA. Patients with chronic pain or opioid use exceeding 30 days and those undergoing surgeries other than primary TKA were excluded. Outcome data, including opioid use (from which post-operative oral morphine equivalents (OME) were calculated), antiemetic use, visual analog pain scale (VAS) scores, distance ambulated at 24 hours, and length of hospital stay, were extracted by chart review.

Results

In the post-anesthesia care unit (PACU), patients in both ITM groups experienced significantly lower opioid consumption and pain scores compared to the control group (p<.001). Furthermore, cumulative OME at 24 hours was significantly less in the ITM groups compared to the control, but there was no difference between ITM doses (p=0.004; mean cumulative OME for control was 77.2 OME vs 43.4 OME for 100 mcg ITM vs 42.6 OME for 150 mcg ITM). Antiemetic usage did not increase in the ITM groups. Although there was no statistically significant difference in ambulation at 24 hours, both ITM groups exhibited a trend toward greater average ambulation distance compared to the control group (p=0.095; mean distance walked for control was 67.6 feet, 76.6 feet for 100 mcg ITM vs 98.8 feet for 150 mcg ITM). Hospital length of stay did not significantly differ between the groups.

Conclusion

ITM doses of 100 mcg and 150 mcg provide effective analgesia for patients undergoing lower extremity total knee arthroplasty under spinal anesthesia. Patients receiving ITM had better pain scores in the immediate post-operative period and had overall less oral morphine equivalent consumption when compared to control. In addition, the safety and side effect profile for ITM is similar for both doses as there was no incidence of respiratory depression and antiemetic usage did not differ between all study arms. Future studies should explore the use of higher ITM doses and consider a broader patient population to further understand the advantages and potential drawbacks of ITM in TKA surgery.

## Introduction

Osteoarthritis (OA), a debilitating and life-altering condition, presents an escalating health challenge as populations in the United States and other developed nations experience increased life expectancy and body mass index (BMI) [1-3]. Among the treatment modalities for end-stage, tri-compartmental, degenerative OA of the knees, total knee arthroplasty (TKA) is a common and cost-effective orthopedic surgical intervention [4, 5]. However, TKA is associated with significant postoperative pain, which can be challenging to manage [6-9]. Effective control of postoperative pain is imperative for successful rehabilitation and overall enhanced recovery [2].

While peripheral nerve blockade (PNB) has been suggested as the optimal solution for effective pain control with a minimized side-effect profile, the use of intrathecal morphine (ITM) has not been thoroughly compared in this context [2, 10]. The PROSPECT meta-analysis demonstrated the superiority of ITM over placebo but found ITM to be equivalent to single-shot and continuous nerve block catheters, but ITM was associated with worse pruritus and decreased satisfaction [2]. Subsequently, ITM has been reserved for cases where regional anesthesia could not be performed [2-3].

Building upon the findings from our previous study, which demonstrated that 150 mcg of intrathecal morphine (ITM) in patients undergoing TKA under spinal anesthesia (SA) resulted in improved postoperative analgesia and reduced opioid consumption compared to the control group [10], this retrospective study aims to delve deeper into potential differences in postoperative analgesia and complications between control and two different doses of intrathecal morphine. We compared three patient groups undergoing primary TKA and receiving preoperative adductor canal blocks (ACB). These groups were compared with regard to the effects of intrathecal morphine (ITM) doses of 100 mcg and 150 mcg, as well as a control group. The control group received continuous adductor canal blocks (CCACB) without ITM, while the experimental groups received single-dose adductor canal blocks (SSACB) along with either 100 mcg or 150 mcg ITM. A comprehensive set of outcome measures was analyzed, encompassing oral morphine equivalents (OME) over time, pain scores, cumulative OMEs, ambulation distance, antiemetic use, and length of hospital stay.

We hypothesized that the combination of SSACB plus SA with ITM (100 mcg) would be non-inferior to that of SSACB plus SA with ITM (150 mcg) alone in regard to postoperative pain, side effects, and patient satisfaction while simultaneously improving practice efficiency. To test our hypothesis we conducted this retrospective study of our TKA patient population, analyzing pre- and post-implementation data for different doses of ITM.

## Materials and methods

Study approval and patient selection

This retrospective study received approval from the Institutional Review Board (IRB) at the University of Alabama at Birmingham. The study focused on three distinct patient groups who underwent primary total knee arthroplasty (TKA) and received preoperative adductor canal blocks (ACB). The control group comprised TKA patients who received continuous adductor canal blocks (CCACB) under spinal anesthesia without intrathecal morphine. In contrast, the experimental groups included TKA patients who underwent single-dose adductor canal blocks (SSACB) under spinal anesthesia, with two different intrathecal morphine dosages - 100 mcg or 150 mcg.

Procedure details

Patients provided informed consent for the ACB block, and the block site was verified during the time-out procedure. Both the control and experimental groups received 50-100 mcg of intravenous fentanyl for sedation during the block. Patients were placed in the supine position, and a high-frequency linear probe was used to identify the target. A 25 ml bolus of 0.5% ropivacaine was administered for the adductor canal block in all three groups. Following the bolus, the CCACB group had a nerve block catheter inserted. All groups received intraoperative periarticular local anesthetic infiltration by the surgeon for TKA. Postoperatively, the CCACB group received a continuous infusion of 0.2% ropivacaine at a rate of 6 ml per hour.

Patient selection

A random selection process was employed, utilizing a random numbers generator to choose 25 patients from the pool of TKA patients in each group. Inclusion criteria encompassed adult patients aged 18 years or older who had undergone primary TKA. For pain management, rescue medication was administered orally, either as oxycodone or hydrocodone, and oral morphine equivalents (OME) were calculated to facilitate comparisons between groups. Exclusion criteria included a medical history of chronic pain or chronic opioid use exceeding 30 days, as well as patients undergoing surgeries other than primary TKA.

Data collection, statistical analysis, and data presentation

Authorized personnel conducted a chart review in the medical record to extract outcome data for this retrospective study per IRB-approved protocol. Data are presented as the mean and standard error of the mean for continuous variables or number and percentage of total for categorical variables. Group comparisons were conducted using either ANOVA with Tukey’s multiple comparison test for parametric data or Kruskal-Wallis with Dunn’s multiple comparison tests for nonparametric endpoints. A p-value of less than 0.05 was considered statistically significant. All statistical analyses and graph generation were carried out using GraphPad Prism version 9.3.1 for Mac OS X (GraphPad, Boston, MA).

Manuscript preparation and figure generation

**Figure 1:**
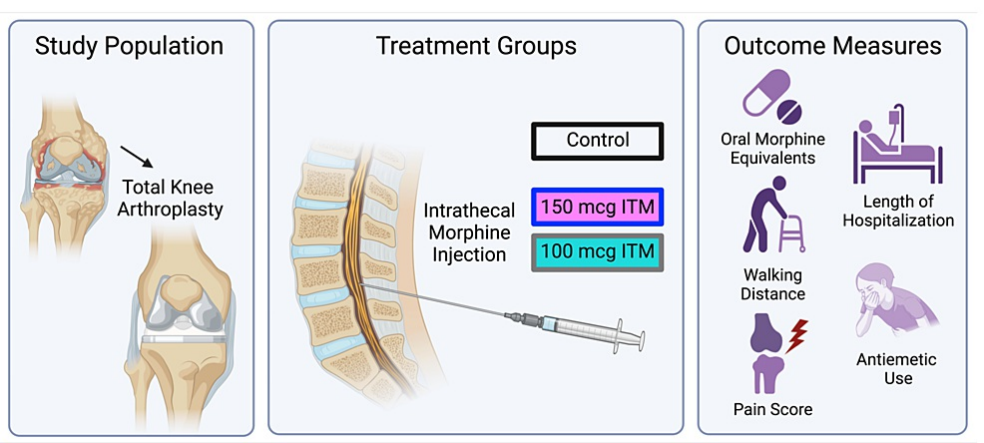
Schematic for this retrospective study which compares intrathecal morphine (ITM) doses in patients undergoing total knee arthroplasty (TKA) under spinal anesthesia. Figure created using Biorender.com.

Figure 1 graphic was created with Biorender.com. Manuscript originally drafted in Microsoft Word Processor (Microsoft, Redmond, WA). Large language model tools (OpenAI, San Francisco, CA; Grammarly, San Francisco, CA) were used for assistance in the editing stage of manuscript preparation.

## Results

Our retrospective study compared three distinct patient groups who underwent primary total knee arthroplasty (TKA) and received preoperative adductor canal blocks (ACB), specifically comparing the effects of 100 mcg or 150 mcg intrathecal morphine (ITM) doses with control. The control group was TKA patients who received continuous adductor canal blocks (CCACB) under spinal anesthesia without intrathecal morphine. The experimental groups were TKA patients who underwent single-dose adductor canal blocks (SSACB) under spinal anesthesia, with either 100 mcg or 150 mcg dose of ITM. Outcome measures for this study were the amount of oral morphine equivalents used (over time and cumulative in 24 hours following surgery), length of time from surgery end to discharge, antiemetic use, visual analog pain scale (VAS) scores during admission, distance ambulated at 24 hours, and length of hospital stay, extracted by chart review (see Figure 1 for schematic overview; Table 1 for patient demographics).

**Table 1:** Patient demographics

Characteristic	Control (n = 25)	100 mcg morphine (n = 25)	150 mcg morphine (n = 25)
Age (years), mean (SE)	67.28 (1.93)	65.56 (1.61)	60.72 (2.32)
Race/Ethnicity, N (%)			
African American	7 (28%)	8 (32%)	14 (56%)
American Indian	1 (4%)	0 (0%)	0 (0%)
Asian	0 (0%)	1 (4%)	0 (0%)
Hispanic/Latino	1 (4%)	0 (0%)	1 (4%)
White	16 (64%)	16 (64%)	10 (40%)
Sex, N (%)			
Female	18 (72%)	16 (64%)	21 (84%)
Male	7 (28%)	9 (36%)	4 (16%)

Outcome measures for the three groups for this study (control, 100 mcg ITM, and 150 mcg ITM) included oral morphine equivalents (OME) as well as pain score in the post-anesthesia care unit (PACU), at 6, 12, and 24 hours post-TKA operation, cumulative OMEs during 24 hours post-operation, feet ambulated during the physical therapy evaluation at 24 hours, use of antiemetics in PACU, and total length of stay in hours from surgery end to discharge (Table 2).

**Table 2:** Outcome measures PACU: Post-anesthesia care unit; OME: Oral morphine equivalent

Outcome	Control (n = 25)	100 mcg morphine (n = 25)	150 mcg morphine (n = 25)	P*
OME, mean (SE)				
PACU	34.26 (6.49)	3.40 (1.16)	1.50 (1.06)	< 0.0001
6 hours	10.54 (2.76)	9.73 (1.67)	4.10 (1.30)	0.054
12 hours	11.19 (2.33)	8.50 (1.64)	7.80 (1.42)	0.392
24 hours	22.38 (3.67)	21.81 (3.53)	27.99 (5.21)	0.520
24 hours (cumulative)	77.21 (10.28)	43.44 (5.90)	42.59 (7.33)	0.004
Pain score, mean (SE)				
PACU	5.92 (0.72)	1.04 (0.53)	0.16 (0.16)	< 0.0001
6 hours	4.28 (0.64)	3.18 (0.79)	1.80 (0.59)	0.017
12 hours	4.00 (0.77)	3.78 (1.04)	3.52 (0.69)	0.869
24 hours	5.43 (0.71)	5.71 (0.59)	6.00 (0.76)	0.853
Ambulation distance at 24 hours (feet), mean (SE)	67.56 (12.91)	76.61 (13.38)	98.88 (11.82)	0.095
Use of antiemetic, N (%)	8 (32.0%)	12 (48.0%)	7 (28.0%)	0.153
Length of stay (hours), mean (SE)	37.96 (5.42)	47.38 (7.78)	33.20 (3.45)	0.175

**Figure 2:**
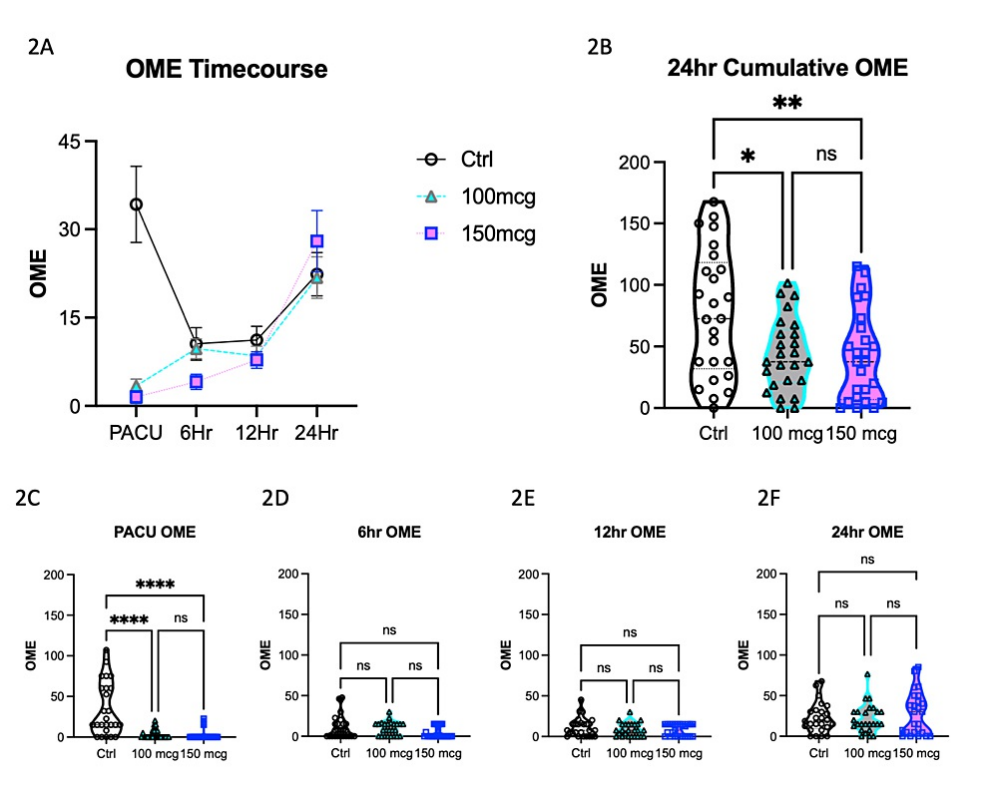
Oral morphine equivalent (OME) by group. (2A) OME utilization over time by group. (2B) Cumulative OME usage is reduced in ITM groups compared at 24 hours postoperatively. OMEs used while in the (2C) PACU, and at (2D) 6 hours, (2E) 12 hours, and (2F) 24 hours following TKA. PACU: Post-anesthesia care unit; TKA: Total knee arthroplasty; ITM: Intrathecal morphine.

To evaluate the analgesic efficacy of ITM and investigate potential dose-related differences, we performed an analysis of the electronic Medication Administration Record (MAR) to examine the distribution of pro re nata (PRN) opioid medication over time (Figure 2). Oral morphine equivalents (OMEs) were calculated for patients during their stay in the post-anesthesia care unit (PACU) and at 6, 12, and 24 hours postoperatively. Figure 2A graphically illustrates the OME time course for each group.

Furthermore, the computation of cumulative OMEs within the initial 24 hours postoperatively revealed significant differences. Tukey's multiple comparison post hoc test confirmed substantially lower cumulative OMEs in both Intrathecal Morphine (ITM) groups when compared to the control group. Specifically, the mean difference between the control group and the 100 mcg ITM group was 33.8 OME (95% CI 6.5-60.9, p=0.01), and the mean difference between the control group and the 150 mcg ITM group was 34.6 OME (95% CI 7.4-62.8, p=0.009). Notably, the mean difference of 0.85 OME between the 100 mcg and 150 mcg ITM groups was not statistically significant (95% CI -26.4-28.1, p=0.99) (Figure 2B).

The analysis of OME within the PACU showed a significant difference among the three groups based on ANOVA, with a p-value of less than 0.0001. Tukey's multiple comparison post hoc test revealed substantial mean differences in OME between the control group and both the 100 mcg and 150 mcg ITM groups. Specifically, the mean difference between the control group and the 100 mcg ITM group was 30.9 OME (95% CI 17.8-43.9, p<0.0001), while the mean difference between the control group and the 150 mcg ITM group was 32.8 OME (95% CI 19.7-45.8, p<0.0001). In contrast, there was no statistically significant difference in OME between the two ITM groups, with a mean difference of 1.9 OME (95% CI -11.2-15.0, p=0.935) (Figure 2C).

OME at the 6-hour post-total knee arthroplasty (TKA) mark did not exhibit a statistically significant difference among the three groups based on ANOVA, although they followed a similar trend as in the PACU (p=0.054). Tukey's multiple comparison post hoc test further confirmed this lack of statistical significance. The mean difference in OME between the control group and both the 100 mcg and 150 mcg ITM groups was not significant, with values of 0.81 OME (95% CI -5.9-7.6, p=0.956) and 6.4 OME (95% CI -0.35-13.2, p=0.067), respectively. Similarly, there was no statistical difference in OME between the two ITM groups, with a mean difference of 5.6 OME (95% CI -1.2-12.4, p=0.124) (Figure 2D).

OME at the 12-hour post-TKA point showed no statistically significant difference across the three groups by ANOVA, with a p-value of 0.948. Tukey's multiple comparison post hoc test revealed no significant mean difference between the control group and both the 100 mcg and 150 mcg ITM groups, with values of 2.7 OME (95% CI -3.5-8.9, p=0.558) and 3.3 OME (95% CI -2.8-9.6, p=0.397), respectively. Additionally, there was no statistical difference in OME between the 100 and 150 mcg ITM groups, with a mean difference of 0.7 OME (95% CI -5.5-6.9, p=0.960) (Figure 2E).

Similar to the 12-hour time point, OME at the 24-hour post-TKA mark did not exhibit a statistically significant difference across the three groups based on ANOVA, with a p-value of 0.659. Tukey's multiple comparison post hoc test confirmed no significant mean difference between the control group and both the 100 mcg and 150 mcg ITM groups. Specifically, the values were 0.57 OME (95% CI -13.7-14.8, p=0.994) and -5.6 OME (95% CI -19.9-8.6, p=0.615), respectively. Likewise, there was no statistical difference in OME between the 100 and 150 mcg ITM groups, with a mean difference of -6.18 OME (95% CI -20.4-8.1, p=0.555) (Figure 2E).

**Figure 3:**
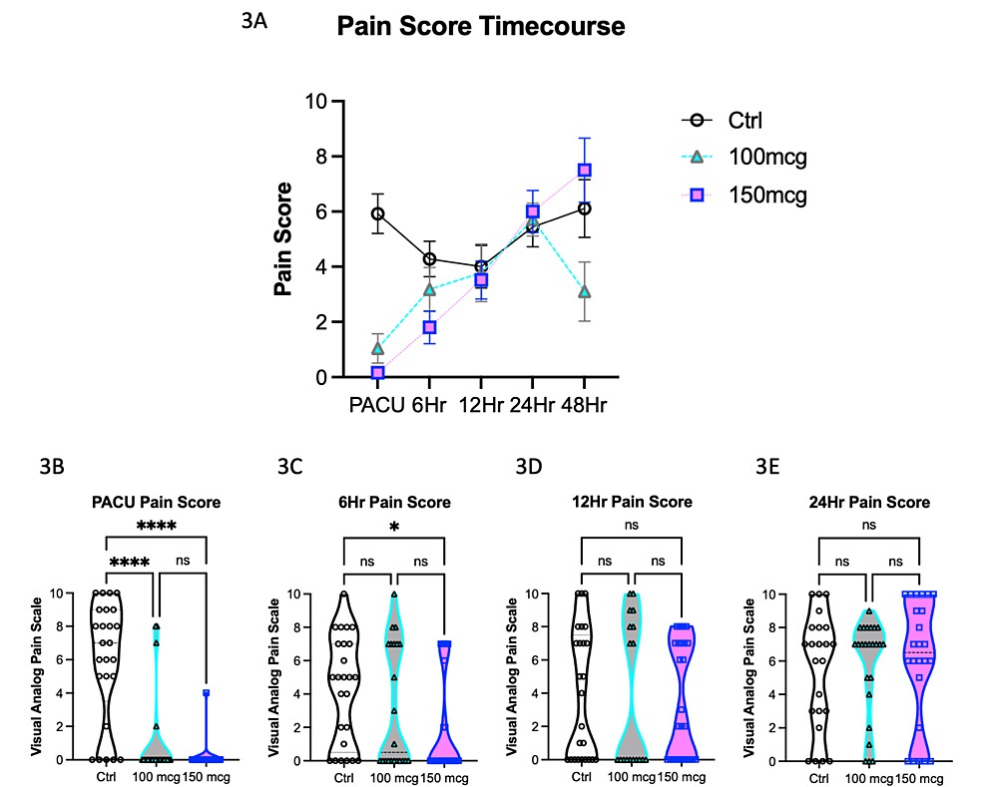
Visual analog pain scale (VAS) scores by group. (3A) VAS scores over time by group. (3B) VAS score is significantly higher in the control group compared to the ITM groups in the PACU, but there is no statistically significant difference between 100 and 150 mcg. (3C) VAS scores at 6 hours post-op are significantly less in the 150-mcg compared to the 100-mcg group. There is no difference in VAS between any group at (3D) 12 hours or (3E) 24 hours. ITM: Intrathecal morphine; PACU: Post-anesthesia care unit.

Visual analog pain scale (VAS) scores were employed to assess subjective pain levels among the three patient groups across different time intervals (Figure 3). Figure 3A presents the VAS scores at various time points for each group.

In the post-anesthesia care unit (PACU), significant differences in VAS pain scores were observed among the groups (Kruskal-Wallis, p<0.0001). As depicted in Figure 3B, the control group exhibited significantly higher VAS scores in comparison to the ITM groups according to Dunn's multiple comparison test. However, no statistically significant difference was found between the 100 mcg and 150 mcg ITM groups. Specifically, the mean rank differences between the control group and the 100 mcg ITM group and the 150 mcg group were 24.5 (p<0.0001) and 29.7 (p<0.0001), respectively. No statistically significant difference in VAS pain scores was identified between the two ITM groups, with a mean rank difference of 4.7 (p>0.999) (Figure 3B).

At the 6-hour postoperative mark (Figure 3C), VAS scores displayed statistically significant differences (Kruskal-Wallis, p=0.02). Subsequent post hoc analysis using Dunn's multiple comparison tests revealed that VAS pain scores were significantly higher in the control group when compared to the 150 mcg ITM group. The mean rank difference was 15.4 (p=0.016). Nevertheless, at the 12-hour (Figure 3D) and 24-hour (Figure 3E) postoperative time points, no statistically significant differences in VAS scores were observed among the groups. Pain levels appeared to converge during these later time intervals, with no significant distinctions.

In summary, the analysis of VAS scores demonstrated variations in pain levels during the early postoperative period, with the control group experiencing higher pain scores in the PACU compared to the ITM groups. Additionally, a significant difference was observed between the control and 150 mcg ITM groups at the 6-hour mark. However, at later time points, VAS scores converged, and no significant differences were noted among the groups, indicating a stabilization of pain perception during the first 24 hours of the recovery period.

**Figure 4:**
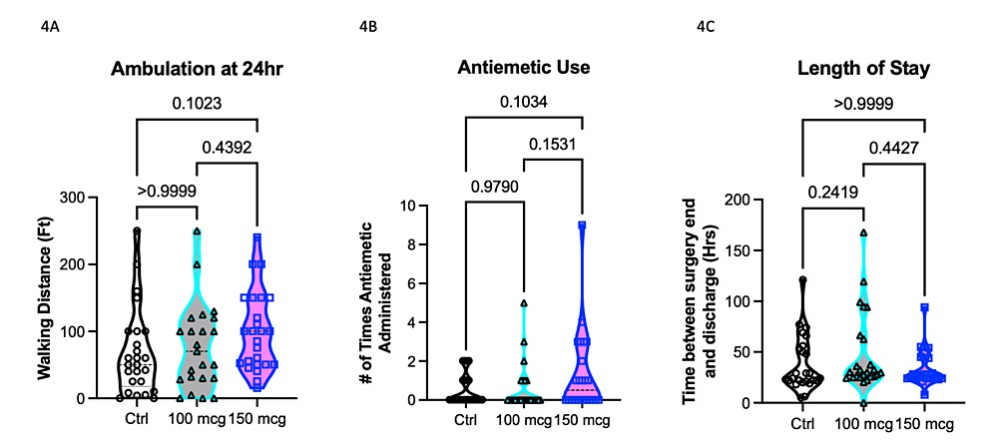
(4A) Ambulation distance at 24 hours, (4B) antiemetic use, and (4C) length of hospitalization from time of surgery end to discharge are not statistically different across groups.

Figure 4 summarizes secondary outcomes related to ambulation distance, antiemetic use, and length of hospitalization for the three study groups. Our hypothesis was centered on the potential effects of varying intrathecal morphine (ITM) dosages on these parameters. We postulated that a higher ITM dose could enhance postoperative analgesia, potentially leading to increased ambulation distances. However, we also anticipated that higher doses might be associated with an elevated risk of nausea, requiring more frequent use of antiemetics, and potentially influencing the length of hospitalization.

Ambulation distance at the 24-hour mark (Figure 4A) did not reveal any statistically significant differences among the three groups, as determined by ANOVA (p=0.19). Additionally, Tukey's multiple comparison post hoc test did not yield statistically significant results. Despite this, a noticeable trend was observed, suggesting that patients in the 150 mcg ITM group tended to walk greater distances compared to the control group. The mean difference between the control group and the 100 mcg ITM group was -9.1 feet (95% CI -52.4-34.3, p>0.99), and the mean difference between the control group and the 150 mcg ITM group was -31.3 feet (95% CI -73.7-11.1, p=0.10). Notably, the mean difference of -22.7 feet between the 100 mcg and 150 mcg ITM groups was not statistically significant (95% CI -65.6-21.0, p=0.44) (Figure 4A).

Analysis of antiemetic use in the post-anesthesia care unit (PACU) (Figure 4B) did not reveal statistically significant differences between the groups (p=0.15). However, a discernible trend was noted, indicating increased antiemetic use in the 150 mcg ITM group compared to the control group (p=0.10). Moreover, the 150 mcg group displayed higher antiemetic use compared to the 100 mcg ITM group (p=0.15).

Figure 4C illustrates the length of hospitalization from the time of surgery completion to discharge. This parameter also did not exhibit statistically significant differences among the groups (p=0.17).

While these results did not achieve statistical significance, the observed trends are noteworthy, suggesting potential clinical implications. It appears that a higher ITM dosage may be associated with increased ambulation due to improved pain control. Simultaneously, there may be a greater likelihood of experiencing nausea, leading to the administration of antiemetics, and a potential impact on the length of hospitalization. These trends, although not statistically confirmed, merit further investigation and consideration due to their potential clinical and economic significance.

## Discussion

Intrathecal morphine (ITM) provides optimal analgesia in patients undergoing total lower limb arthroplasty surgery. In this retrospective study, we compared two doses (100 mcg and 150 mcg) of ITM, however, the analgesia benefits of ITM did not differ between the two ITM groups. In the immediate postoperative period, as observed in the post-anesthesia care unit (PACU), both ITM groups displayed lower oral morphine equivalents (OME) and pain scores when contrasted with the control group (p<.001). There was no significantly different usage of antiemetics. There was not an increased usage of antiemetics in any treatment group. Although there was not a statistically significant difference in ambulation at 24 hrs, there was a trend toward farther ambulation in the ITM groups compared to the control (p=0.09; mean distance at 24 hours for control 67.6 ft, SEM 12.9 vs 76.6 ft, SEM 48.9 SEM for 100 mcg vs 98.9 ft, SEM 11.82 for 150 mcg ITM). Overall hospital length of stay did not differ in the three groups.

Inadequately controlled pain after surgery can hinder early mobilization. The use of multimodal analgesic therapy has been shown to decrease acute post-op pain and reduce post-op opioid consumption in various surgeries. There are many benefits to the addition of ITM. It has a long duration of action, promotes early mobilization, and decreases hospital stay [11]. Unlike IT local anesthetics, ITM does not cause muscle weakness, sympathectomy, or loss of proprioception. When compared to parenteral opioids, neuraxial opioids have been found to provide better analgesia and less opioid consumption in certain surgeries [12].

Many studies have shown the administration of ITM provides post-operative pain relief in orthopedic procedures [11, 13-18]. One of our aims was to examine the analgesic effects of various ITM doses. Sibanyoni et al. examined the analgesic effect of 150 mcg ITM vs 100 mcg ITM dose in patients undergoing lower limb arthroplasty surgery [19]. They concluded that the 150 mcg ITM dose was superior and the side effect profile with the higher dose was comparable to the lower dose group. Hassett et al. demonstrated a poor analgesia effect of 100 mcg ITM when compared to higher doses of ITM [15]. In our study, we did not find a significant difference in analgesic effects and OMEs between the 100 mcg and 150 mcg ITM group at all recorded time points. At the post 6 hr mark, the pain score was the lowest for the 150 mcg group (p<.017). At 24 hr, the cumulative OME was less in both ITM groups (43.33 in the 100 mcg ITM vs 42.59 in the 150 mcg ITM) when compared with the control (77.21).

The literature has mixed results on what is the optimal ITM dose. Many dose-response studies found ITM optimal dosing ranges from 0.075-0.15 mg and doses higher than 0.15 mg ITM were associated with more side effects including pruritus, nausea, and vomiting. The 150 mcg ITM group had a high percentage of patients who had nausea and vomiting compared to the 100 mcg ITM group. However, we did not notice a difference in the use of antiemetics in each group as compared to each other and also when compared with the control group.

When utilizing ITM, there is concern for delayed respiratory depression, which has been reported to occur between 6 and 18 hours after injection [20]. We did not report any incidence of respiratory depression. Risk factors for ITM-induced respiratory depression include increased age, long-acting sedatives, co-existing respiratory disease, and chronic opioids. Various subspecialty societies have devised respiratory monitoring recommendations - Society of Obstetric Anesthesia and Perinatology for the obstetric patient undergoing cesarean delivery and American Society of Anesthesiologists and American Society of Regional Anesthesia and Pain Medicine for the general surgical patient population [21, 22].

The incidence of respiratory depression secondary to ITM is unknown, as many studies have lacked a standard definition for respiratory depression. In this retrospective study, we defined respiratory depression as the need for medical emergency response team evaluation. Retrospective and prospective analyses of mixed populations (obstetric and non-obstetric) indicate an incidence of respiratory depression ranging from 0.26% to 3% for ITM doses ranging from 0.15 to 0.8 mg [22]. ITM doses greater than 0.25 mg may be associated with a higher incidence of respiratory depression [22]. The safety profile for the use of low-dose ITM (≤150 mcg) for analgesia is extremely favorable.

There are several limitations of this study, one being the retrospective nature of the study. It will be challenging to conduct a prospective study to include higher doses of ITM based on patient safety and ethical issues. Second, although our study shows favorable and comparable analgesic outcomes with the use of different ITM doses, these results may not be generalizable. Third, the study population included patients undergoing lower extremity total knee arthroplasty and the outcome results may not translate to a different surgical patient population or age group. Fourth, recruitment into study arms did not undergo matching. There was a higher proportion of females and Caucasians in this study. Future studies with a big sample size are warranted to better understand the benefits and adverse effects of commonly used ITM doses for total joint arthroplasty surgery under spinal anesthesia.

## Conclusions

This study demonstrated that the use of ITM, regardless of dosage, resulted in significantly lower OMEs and pain scores in the immediate postoperative period, particularly in the post-anesthesia care unit (PACU), compared to the control group. Notably, there were no statistically significant differences between the two ITM groups, indicating that both doses provided comparable analgesia. The findings suggest that a higher ITM dose may not necessarily translate to improved ambulation or reduced hospital stay, while indicating the safety and efficacy of lower ITM doses (≤150 mcg) for pain management in total knee arthroplasty surgery. This study adds to the growing body of evidence supporting the use of ITM in orthopedic procedures. The study acknowledges that the specific patient population and surgical context may influence the optimal ITM dosage, highlighting the need for further research in different surgical populations.
